# Supplementary figures and images for: Fire severity effects on resprouting of subtropical dune thicket of the Cape Floristic Region
Source: PeerJ. 2020 Jun 10;8:e9240. doi: 10.7717/peerj.9240 (PMC7293192; doi:10.7717/peerj.9240)

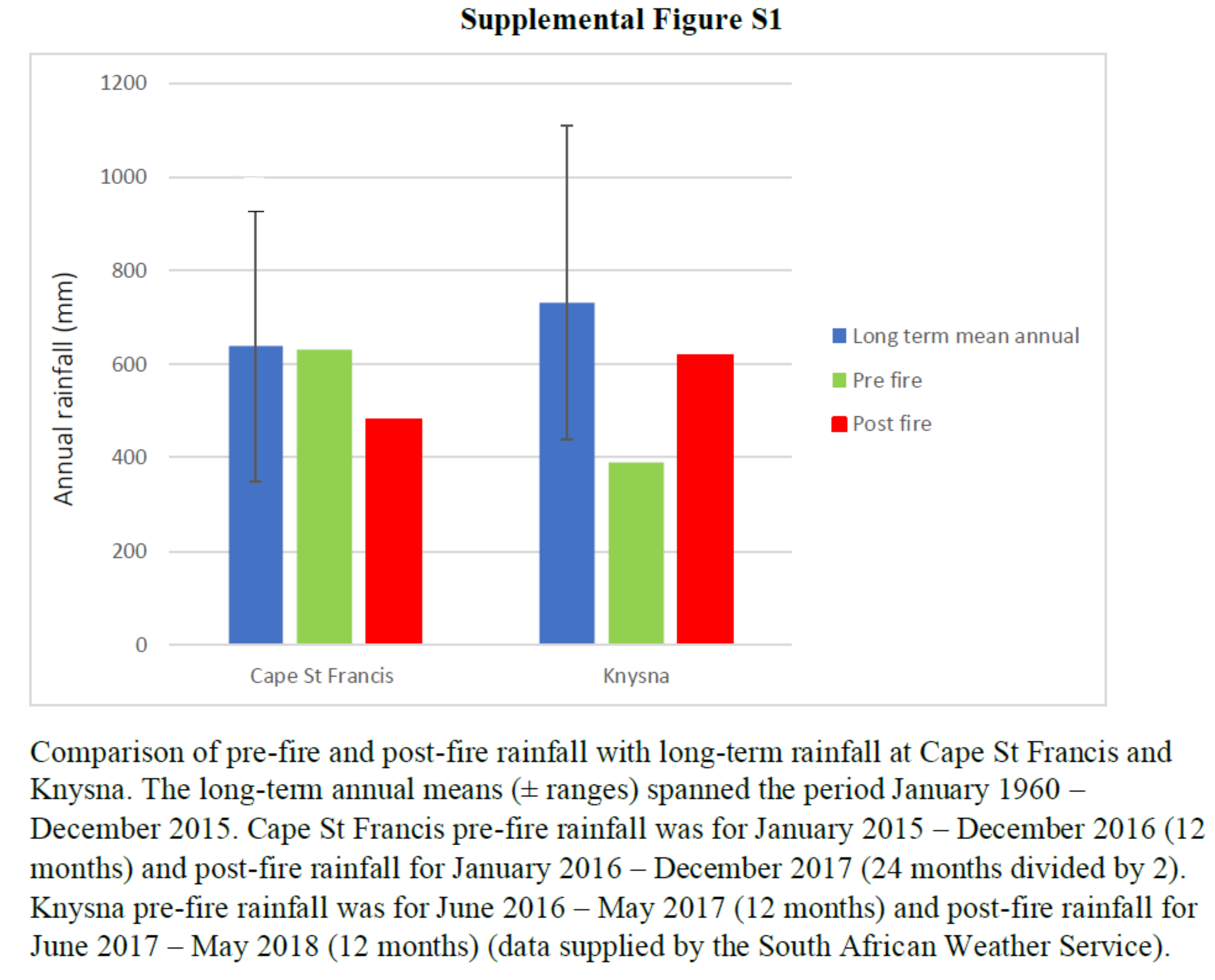

Supplement: Supplemental Information 1 [file peerj-08-9240-s001.png]

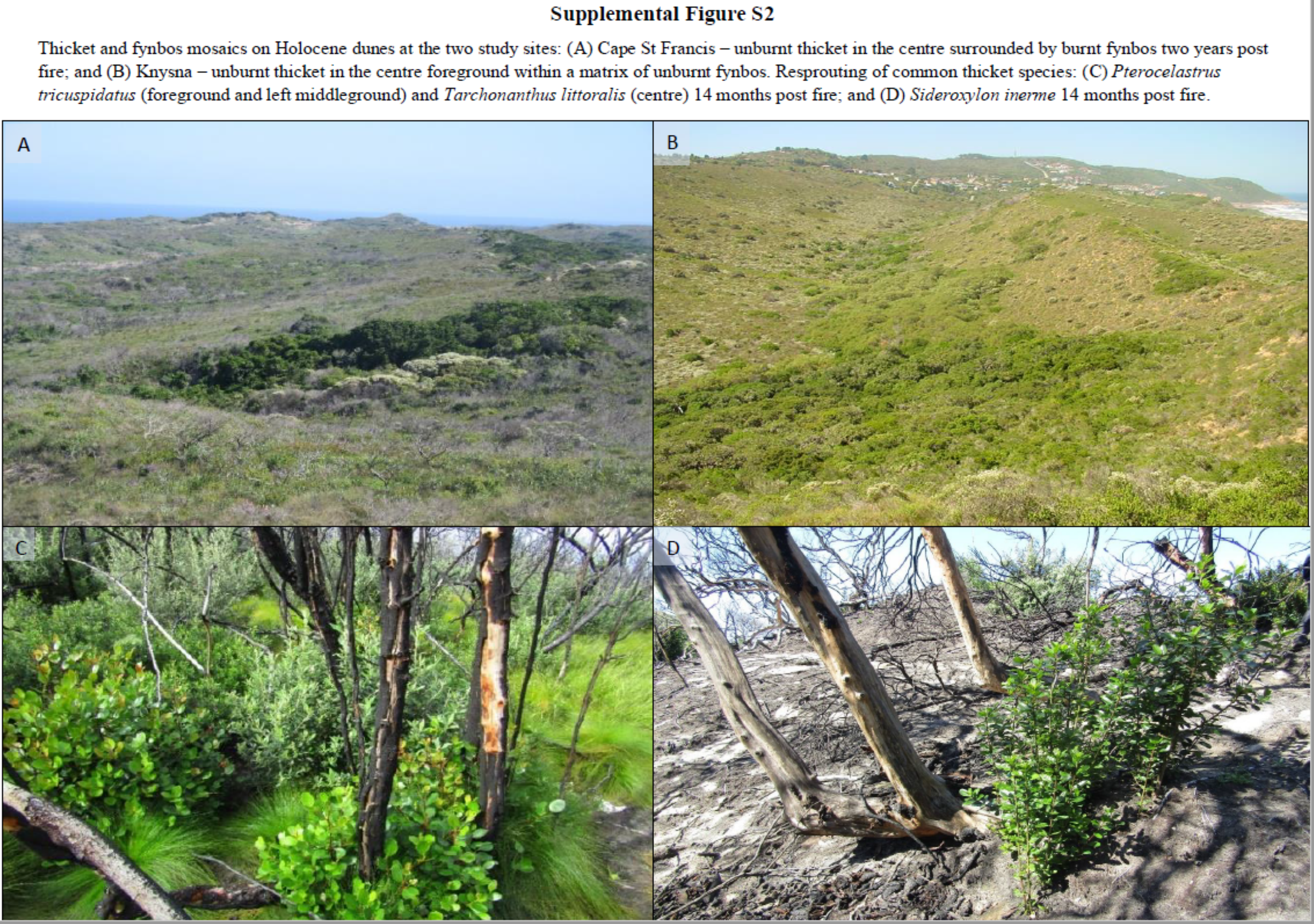

Supplement: Supplemental Information 2 [file peerj-08-9240-s002.png]

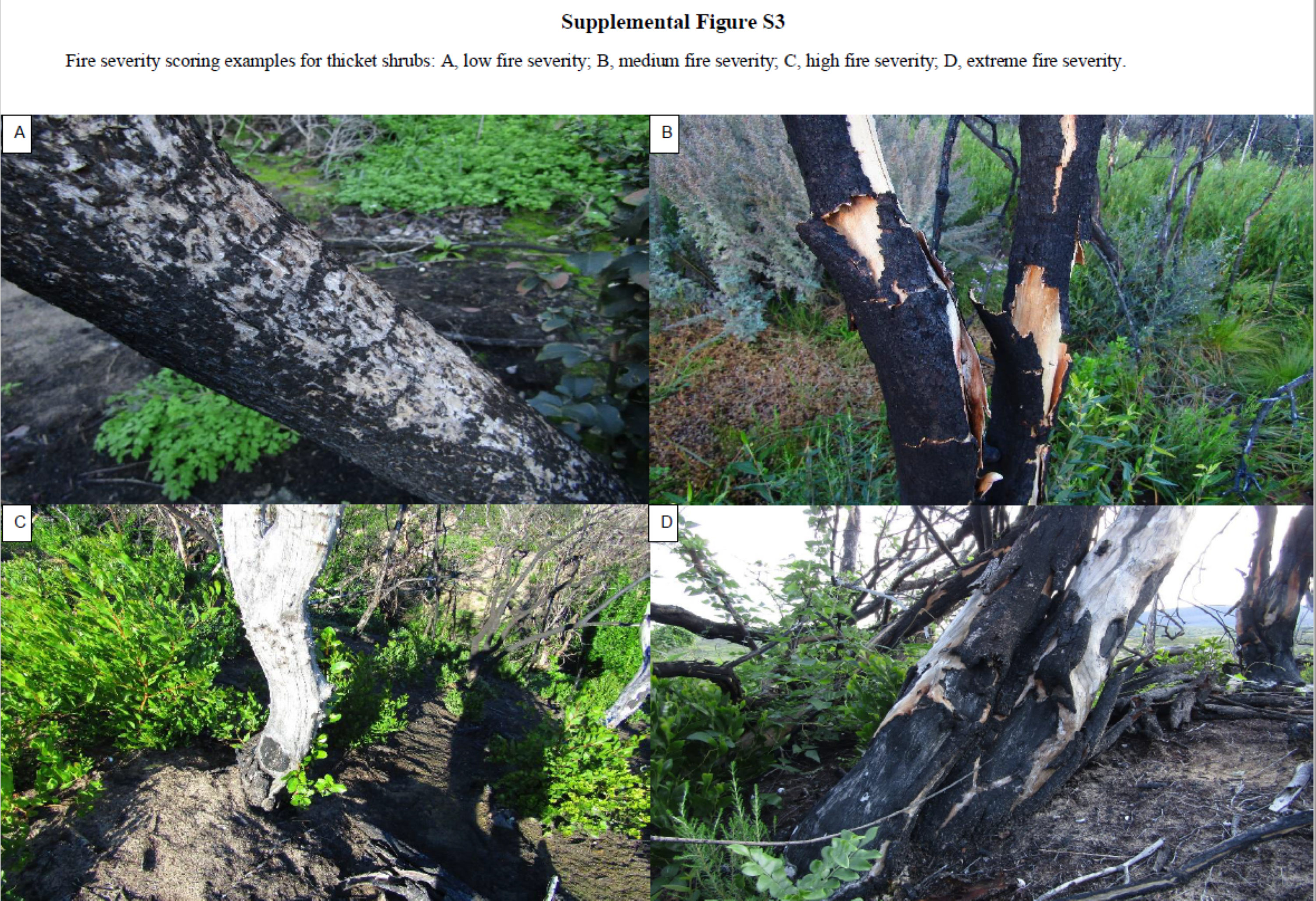

Supplement: Supplemental Information 3 [file peerj-08-9240-s003.png]

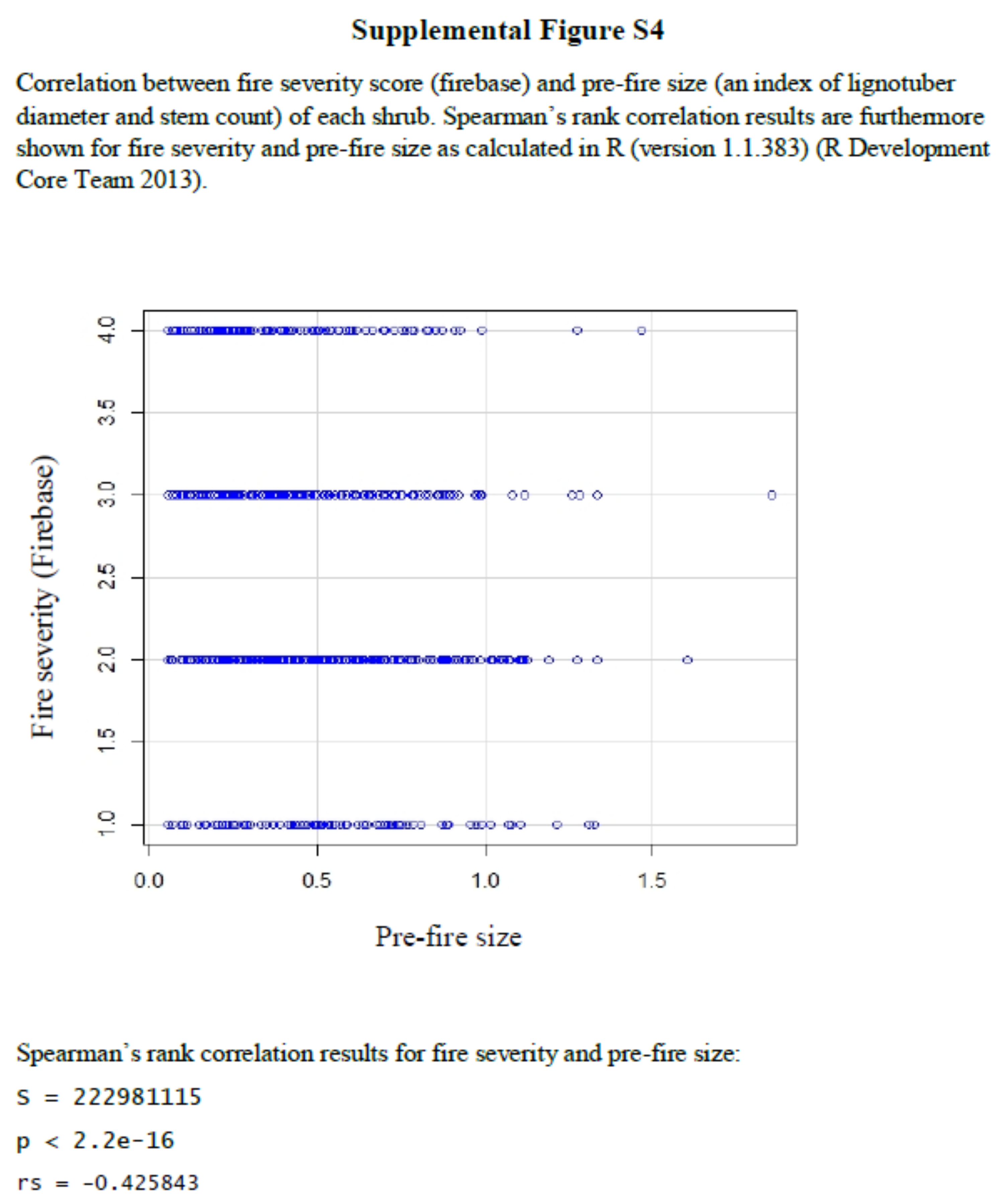

Supplement: Supplemental Information 4 [file peerj-08-9240-s004.png]
