# Supplementary material for: Fire severity effects on resprouting of subtropical dune thicket of the Cape Floristic Region
Source: PeerJ. 2020 Jun 10;8:e9240. doi: 10.7717/peerj.9240 (PMC7293192; doi:10.7717/peerj.9240)
Supplement: Supplemental Information 5 [file peerj-08-9240-s005.pdf]

Supplemental Table S1

The Lowveld Fire Danger Index (FDI-W)[A,B,C] prior to and days of fire (shaded in grey) for Cape St Francis[D] and Knysna.

Cape St Francis[D]			Knysna		
Date	Category	Description	Date	Category	Description
01.01.16	48	Dangerous	11.05.17	46	Dangerous
01.01.16	49	Dangerous	12.05.17	35	Moderate
03.01.16	51	Dangerous	13.05.17	37	Moderate
04.01.16	49	Dangerous	14.05.07	49	Dangerous
05.01.16	47	Dangerous	15.05.17	50	Dangerous
06.01.16	50	Dangerous	16.05.17	65	Very dangerous
07.01.16	25	Moderate	17.05.17	41	Moderate
08.01.16	14	Safe	18.05.17	41	Moderate
09.01.16	25	Moderate	19.05.17	51	Dangerous
10.01.16	52	Dangerous	20.05.17	46	Dangerous
11.01.16	63	Very dangerous	21.05.17	47	Dangerous
12.01.16	51	Dangerous	22.05.17	51	Dangerous
13.01.16	43	Dangerous	23.05.17	67	Very dangerous
14.01.16	47	Dangerous	24.05.17	53	Dangerous
15.01.16	19	Safe	25.05.17	23	Moderate
16.01.16	60	Dangerous	26.05.17	25	Moderate
17.01.16	61	Very dangerous	27.05.17	23	Moderate
18.01.16	58	Dangerous	28.05.17	51	Dangerous
19.01.16	49	Dangerous	29.05.17	48	Dangerous
20.01.16	53	Dangerous	30.05.17	59	Dangerous
21.01.16	45	Moderate	31.05.17	55	Dangerous
22.01.16	43	Moderate	01.06.17	40	Moderate
23.01.16	44	Moderate	02.06.17	51	Dangerous
24.01.16	27	Moderate	03.06.17	90	Extremely dangerous
25.01.16	29	Moderate	04.06.17	38	Moderate
26.01.16	31	Moderate	05.06.17	47	Dangerous
27.01.16	43	Moderate	06.06.17	85	Extremely dangerous
28.01.16	50	Dangerous	07.06.17	84	Extremely dangerous
29.01.16	55	Dangerous	08.06.17	38	Moderate
30.01.16	55	Dangerous	09.06.17	32	Moderate
31.01.16	51	Dangerous	10.06.17	70	Extremely dangerous
01.02.16	84	Extremely dangerous	11.06.17	35	Moderate
Mean	46	Dangerous	Mean	47	Dangerous

[A]Meikle, S., and J. Heine. 1987. A fire danger index system for the Transvaal Lowveld and adjoining escarpment areas. South African Forestry Journal 143: 55-56

[B]The Lowveld Fire Danger Index Category is as follow; 0-20, Safe; 21-45, Moderate; 46-60, Dangerous; 61-75, Very dangerous; 75<, Extremely dangerous.

[C]Data supplied by the South African Weather Service.

[D]The Fire Danger Index for Cape St Francis is based on the FDI for Port Elizabeth which is 70 km apart.
