# Supplementary material for: Fire severity effects on resprouting of subtropical dune thicket of the Cape Floristic Region
Source: PeerJ. 2020 Jun 10;8:e9240. doi: 10.7717/peerj.9240 (PMC7293192; doi:10.7717/peerj.9240)
Supplement: Supplemental Information 6 [file peerj-08-9240-s006.pdf]

Supplemental Table S2

Analysis of Deviance Table (Type II tests) (summarised in the main text in Table 2, 3 and 4) for survival, post-fire resprouting shoot count and post-fire resprouting volume.

**Survival**	## Response: Survival<br>## LR Chisq Df Pr(>Chisq)<br>## Firebase 24.1085 3 2.371e-05 ***<br>## Prefiresize 26.2579 1 2.987e-07 ***<br>## Site 4.9415 1 0.0262191 *<br>## Firebase:Prefiresize 16.3321 3 0.0009694 ***
**Resprouting shoot count**	## Response: Resprouting shoot count<br>## LR Chisq Df Pr(>Chisq)<br>## Firebase 12.030081 3 7.2808e-03 ***<br>## Prefiresize 48.777106 1 2.8677e-12 ***<br>## Site 0.014424 1 9.0441e-01 ***<br>## Firebase:Prefiresize 5.662530 3 1.2923e-01 ***<br>## Firebase:Site 2.031010 3 5.6600e-01 ***<br>## Prefiresize:Site 27.775685 1 1.3623e-07 ***<br>## Firebase:Prefireszie:Site 12.770054 3 5.1612e-03 ***
**Resprouting volume**	## Response: Resprouting volume<br>## LR Chisq Df Pr(>Chisq)<br>## Firebase 34.169 3 1.8252e-07 ***<br>## Prefiresize 55.422 1 9.7256e-14 ***<br>## Site 31.969 1 1.5666e-08 ***<br>## Firebase:Site 55.569 3 5.1927e-12 ***<br>## Prefiresize:Site 19.495 1 1.0086e-05 ***
