# Supplementary material for: Fire severity effects on resprouting of subtropical dune thicket of the Cape Floristic Region
Source: PeerJ. 2020 Jun 10;8:e9240. doi: 10.7717/peerj.9240 (PMC7293192; doi:10.7717/peerj.9240)
Supplement: Supplemental Information 7 [file peerj-08-9240-s007.pdf]

Supplemental Table S3

Soil nutrient differences between dune fynbos, thicket and forest (Cowling 1984).

Community	pH	Sand	S-value	Exchangeable Ca	Oxidizable Carbon	Total N	Available P	n
Fynbos	7.0	96	14.9	2408	6.2	0.25	19.3	3
Fynbos-Thicket	7.5	99	14.4	2454	7.3	0.25	29.6	5
Thicket	7.4	99	15.4	2330	6.7	0.24	20.4	5
Forest	7.2	99	18.4	2151	6.5	0.26	10.7	1
