# Supplementary material for: Fire severity effects on resprouting of subtropical dune thicket of the Cape Floristic Region
Source: PeerJ. 2020 Jun 10;8:e9240. doi: 10.7717/peerj.9240 (PMC7293192; doi:10.7717/peerj.9240)
Supplement: Supplemental Information 8 — Survival = logistic regression model (binomial family and logit link function). [file peerj-08-9240-s008.pdf]

Supplemental Code S1

Formulae used in R (version 1.1.383) (R Development Core Team 2013) to assess survival for dune thicket shrubs. Survival = logistic regression model (binomial family and logit link function).

**Survival**	`modS1g <- glm(Survival ~ Firebase * Prefiresize + Site, family = binomial(),`<br>`data = transform(TSurv, Firebase = factor(Firebase, ordered = T)))`
