# Supplementary material for: Fire severity effects on resprouting of subtropical dune thicket of the Cape Floristic Region
Source: PeerJ. 2020 Jun 10;8:e9240. doi: 10.7717/peerj.9240 (PMC7293192; doi:10.7717/peerj.9240)
Supplement: Supplemental Information 9 — Resprouting shoot count = generalised additive model for location, scale and shape (Delaporte distribution, logarithmic link function). [file peerj-08-9240-s009.pdf]

Supplemental Code S2

Formulae used in R (version 1.1.383) (R Development Core Team 2013) to assess post-fire resprouting shoot count for dune thicket shrubs. Resprouting shoot count = generalized additive model for location, scale and shape (Delaporte distribution, logarithmic link function).

**Resprouting shoot count**	`gamlssResprouting shoot count <- gamlss(Resprouting shoot count ~ Firebase * Prefiresize * Site, data = TRsp, family = DEL(), n.cyc = 500, trace = F)`
